# Rachel Challice's Vow

**Published:** 1888-02-18

**Authors:** Lina Nevill

**Affiliations:** Author of "A Future on Trust," "Juno," &c.


### Rachel Challice's Vow.

By Lina Nevill, Author of "A Future on Trust," "Juno," &c.

Chapter IX.—A Fresh Case.

"Reason's whole pleasure, all the joys of sense,
Lie in three words, health, peace, and competence."
—*Pope.*

"What time did you say that horrid person of yours was coming, mother?"

"I don't know, exactly, darling. Some time this morning; so the matron of the hospital says in her letter."

"I won't see her when she comes; no earthly persuasion will induce me to do so. I cannot conceive what possessed you to go and hunt up and engage someone to look after me, when you know that I detest strangers in my room. However, it makes no difference, except the waste of your time, and trouble; I will not see her. You can just give her a week's wages, and send her back where she came from."

"But, my darling, you must have someone to look after you properly, or you will never get well; Doctor Graves says so. Price does complain so much that the extra work knocks her up, that I was forced to follow his suggestion, and engage a properly trained nurse for you."

"Doctor Graves is an idiot!" exclaimed Nell Endsleigh impatiently. "Yes, as you say, Price is a little annoying. She has been a perfect martyr lately. I scarcely dare say good morning and ask her how she is when she brings in my early cup of tea. It is always, 'Not much to boast of, thank you, Miss Nell. I haven't closed my eyes the whole of this blessed night.' Still, poor dear Price must have a very lovely character. Now when *I* lie awake a whole night I am much more wicked. I never call it a 'blessed' one; in fact, I fear I anathematise it as something very much the reverse!"

"Never mind, dear, Price will not worry you after to-day. You shall have a nice cheerful new nurse to look after you soon," said Mrs. Endsleigh in a soothing tone.

"It is all very well for you to say that. Some people always take it for granted that what is good for one must be nice. You invariably tried to persuade me when I was a child that rice pudding was infinitely preferable in every way to tipsy cake. I accepted the theory, but refused the practice. I have no doubt the nurse is a perfect pattern, but, privately, I shall not like her nearly so well as if everyone else abused her."

"But at all events you will be saved from Price's complaints."

"Not in the least," returned Nell stoutly. "She developed a new grievance this morning. Now the trial is that she will not be called upon to attend to me any longer. For half an-hour this morning she stood at the foot of my bed, mopping her eyes with her handkerchief, and gasping out between her sobs that 'she did think it hard, after all that she had done, that mistress should go and engage a stranger, as though *she* wasn't fit to nurse me longer!' You see, mother, there is no refuge from Price after all; and of the two things I prefer the grumbling. The handkerchief and sentiment are more than I can endure calmly."

Mrs. Endsleigh smiled.

"I think you can endure anything except not getting your own way. You grow more self-willed every day, Nell."

"It is my only amusement," replied the girl wearily. "I can't do anything else but contradict people; and as you always taught me that whatever I did was worth doing well, I make a point of never agreeing with anyone about anything."

"And succeed too!" returned her mother, looking at Nell with a half-smothered sigh.

She was so altered, even in this short time; so utterly unlike the bright, handsome girl she had been only a fortnight before. She had been one of the beauties of the last Drawing Room, and had begun her season triumphantly by becoming at once engaged to one of the most eligible *partis* of the year—a young baronet whom many mothers coveted as a husband for their daughters. But, seemingly without an effort, Nell Endsleigh bore him off—a girl only just out of the schoolroom; a handsome girl to be sure, but as wild as a hawk, and without a particle of style—at least, so said the vast army of disappointed rival mothers and daughters. And the very fact of her having secured her game so easily and unconsciously made her enemies more rabidly rancorous.

Sir Charles Austin was attracted by Nell the first moment he saw her. She was so perfectly natural, so utterly unaffected. She seemed to take it as a matter of course that he should ask to be introduced to her; and he was piqued to find that she had no intention of giving him dances she had promised to others; generally, the girls he danced with were only too pleased to throw over other less promising partners for him.

Nell gave him her one disengaged dance; and when he asked for a second told him calmly that she had not another free.

"Throw someone over," he suggested, thinking himself sure of her assent.

"Why?" she asked innocently. "I should prefer dancing with them, thank you. There are plenty of other girls you can ask."

So there were, and plenty who would have scrambled for the handkerchief had he thrown it. But he was sulky, and did not choose to dance.

If he could not have Nell Endsleigh he would havenobody. The girl was unconsciously playing her cards more surely and scientifically than if she had studied the game for a whole course of seasons.

Neglect and indifference were the only things the petted young man had never met with; and so piqued and attracted was he by the new treatment, that he underwent it on every possible subsequent occasion; until one afternoon at Hurlingham he made up his mind to ask Nell to marry him. They were sitting together on two chairs under one of the spreading trees beyond the conservatory. The lake-like piece of water and the lawn tennis courts lay before them. There were few other people near; nearly everyone else was looking at the polo, save a few energetic tennis players, and two or three young people, who preferred quietness and each other to any game.

The big umbrellas were set up over the tea tables, and as the minutes flew by people began to gather together on the terrace outside the house, and the waiters had more than they could do to supply the various wants.

"I must see where mother is; I left her with the Lindleys," said Nell presently.

"Wait a few minutes longer," he said humbly, "I so seldom see you quite alone; you are always talking to someone else."

"Surely you would not like me to be dumb, would you, or answer 'yes' or 'no' in an undertone, like the eldest Miss Lindley?" she asked mischievously.

"I should not like you different in any way from what you are," he replied warmly.

"Thank you, how delightful. To one person at least I am flawless. I shall tell mother what you say, but she won't believe it; relations have such an uncomfortable way of exalting your faults above your perfections."

"I would not."

"But then you are not a relation."

"I might be one."

"An unknown cousin seven times removed!" she replied lightly. "See, mother and the Lindleys have settled themselves over there. I am thirsty; I want to have tea at once."

Charley's tone had become dangerously tender, and she wished to put an end to the tête-à-tête."

"You shall go in a moment, Nell," he said hurriedly. "Only give me one word of hope. Will you marry me?"

"I don't know," she said piteously, her self possession for the moment deserting her. "I can't tell, with all these people about. Let me write to you to-morrow."

"Very well," he answered, loving her all the more that she had not flown at him in response to his inquiry; but more than ever resolved Nell Endsleigh, and Nell Endsleigh alone, should ever be his wife.

Her answer came next morning. Nell, driving home from Hurlingham in the quiet evening dusk, found by the time the Fulham Road was reached that she liked Charley Austin very much; and when the carriage drove in through the Park she discovered that she loved him passionately.

A week of happiness followed; and then came her accident. Riding in the Park one morning with a girl friend, and expecting Charley to join them, she would not go beyond the entrance to the Row. Her horse became fidgetty at being restrained when he wished to join the others, and at length, frightened by a passing drag, and chafed and fretted by being held in, he became unmanageable. Nell was a fairly good horsewoman, but not equal to the horse in his present excited state, and when he swerved violently, almost coming into collision with the railing, she was thrown. Sir Charles Austin arrived just in time to see Nell being taken, quite insensible, to her mother's house in Grosvenor Square; and from that day she had never left her room, though the doctors gave every hope of her ultimate recovery.

She was allowed to see Charley once a day for a quarter of an hour. But she found her enforced quiet, cut off from all the friends and gaieties of the season, very depressing. To know that everything was going on just the same; that ball after ball, to which she had been invited, was passing, and that she was not able to go; it needed a more stoical mind than Nell's to bear this patiently. It was her first season, and she was only eighteen.

She grumbled, and, as her mother said laughingly, was as contradictory as it was possible for any mortal to be. Yet on the whole the poor girl bore her trial wonderfully well. After a silence of a few moments, she continued to protest stubbornly that no earthly persuasion should induce her to see the new nurse, much less have anything to do with her.

"Not even if Charley wished it?" asked Mrs. Endsleigh archly.

Nell's pale cheeks became rosily pink.

"Well, perhaps then. I think I may safely agree to any very such remote contingency; for I am quite certain he would never ask me to do anything I did not like."

"No, that is only left for your cross old mother," observed Mrs. Endsleigh a little sadly.

"You are never cross; you are a very superior, dear, old mother, whose only fault is that you don't consider me perfect, as Charley does. You know that is why I promised to marry him, because he said there was no room for improvement in me; I dislike the process of being improved. Still, to show that I don't think you cross, I will consent to see this nurse. Only she must not stay. Promise me, Mother, you won't keep her."

"Not if, after a fair trial, you really dislike her."

"What do you call a fair trial? Two hours?

"A whole week."

"What! You will sacrifice me to the tender mercies of some terrible old harpy for a whole, long week! And remember since my accident the weeks have increased to just double their usual length. I won't have it."

"Please, ma'am, a young person wishes to see you," said a melancholy voice at the door, and Price put her tearful face partly inside the room.

"Come in," cried Nell. "Tell me what sort of a person she is."

"I am sure I can't describe her, miss. I couldn't see much of her. She has a long cloak, and a bonnet and a veil, and she asked for the mistress."

"Oh, it is the new nurse!" exclaimed Nell excitedly, as capriciously eager to see her as before she had been averse to the idea. "Tell me all about her, Price. How old is she?"

"I am sure I can't say, miss," replied Price, with an air of dignified martyrdom."

"I suppose you could make a guess though," said Nell impatiently. "Is she twenty or fifty?"

"Something between the two, miss, very likely."

"Price, you get more stupid every day! Is she as old as you are?"

Price's ever-ready handkerchief went quickly up to her eyes. Her age was a tender point, which she did not wish discussed; so she contrived ingeniously to elude the inquiry.

"I know I am stupid and useless now," she sobbed. "And an interloper is got in to come between me and the young lady who——"

"Price," broke in Mrs. Endsleigh's gentle voice, from the window, "go down and ask the nurse to come up here; you can show her the way."

Price gave an audible sob, and retreated.

Rachel was waiting in a little room on the ground floor, rather overawed by the size and grandeur of the house.

"Come with me, please," said Price, in the dictatorial tones she was rather too much given to use towards the junior servants of the establishment. "My mistress and Miss Endsleigh wish to see you."

Rachel rose at once.

She was bewildered by the beautiful things she was passing—the china, the pictures, the very carpets and curtains. Some of the doors were ajar, and through them she caught glimpses of rooms such as she had read about, but had never seen.

Presently Price stopped before a door, and knocked.

"Come in," cried a young voice, and Rachel found herself before her new charge.

If the rest of the house seemed beautiful, it was nothing compared to the room she now found herself in.

The window looked over the square, and from it could be seen the trees and shrubs swaying slowly backwards and forwards in the gentle summer morning air.

Everthing inside seemed white, with touches of blue here and there, a delicate china blue, that looked cool and refreshing after the hot glare of the streets. There were palms and other plants in large silvery Indian metal pots; and a china bowl filled with yellow roses stood on a small table by a couch. On that couch lay the girl Rachel had come to nurse.

Nell Endsleigh had every right to the reputation she had won for beauty. Her face, though it was pale and thin now, was still handsome, and the small, clear cut features and large brown eyes were as attractive as ever.

She smiled as Rachel came in.

"So you have come to make me well," she said in her frank winning voice. "You must be as quick about it as you can, for I am a very impatient patient."

"I will do my best," replied Rachel, charmed with the gracious unaffected manner that had won Nell so many admirers during her short taste of the London season.

"What are we to call you? We have never heard your name," said Mrs. Endsleigh, leaving her place by the open window, and coming towards Nell's couch.

"Nurse Challice, please," replied Rachel.

Mrs. Endsleigh looked at her critically. She was delighted at the appearance of the young nurse, and liked her gentle voice and manner; she felt instinctively that she could trust her, and leave the care of her darling safely in her hands. Nell too seemed prepossessed in the stranger's favour, and to have forgotton all her former prejudice and dislike to the idea of being nursed by a stranger.

"She is so quiet, and seems to know exactly what I like without my telling her," Nell said to Charley that same afternoon, when he came in for his daily short visit.

"I think I shall give up the idea of marrying you," she continued, "and become a hospital nurse instead."

"Then I shall become a confirmed invalid, and engage you permanently to nurse me."

Nell burst out laughing. There was something so utterly ludicrous in the idea of the tall, athletic young man before her sinking into the condition of a confirmed valetudinarian, a poor creature never without some complaint, to whom a new kind of pain is a melancholy joy; and to be more ill than your neighbour, and have the fact generally accepted, unmitigated bliss.

Charley Austin was the reverse of all this—an active, open-hearted man; devoted to sport in every form, and ready to "rough it" if necessary.

"You look better already, Nell," he said, wistfully, trying to persuade himself that there was a marked improvement in her state since his visit of the day before.

"Of course I do," she replied, cheerfully. "My nature is just the reverse of poor Mrs. Dombey's. My life consists of one succession of efforts; and perseverance, as I was taught in my cradle, is sure of reward in the end."

"I wish it would come a little quicker, though," he responded, dolorously. He was getting rather tired of this indefinite waiting for his bride.

"Ah, but you forget the patience without which the perseverence is nothing. Charley, I must put you through a severe course of study at once; your education must have been disgracefully neglected in the matter of moral proverbs and maxims."

Rachel came in as Sir Charles was leaving; and he held out his hand to her, frankly.

"Miss Endsleigh says you are very good. You must take every care of her, and make her quite well very soon."

"That is Sir Charles Austin. He comes to see me every day. We are engaged to be married. What do you think of him?" said Nell, when he had left the room.

"He is very handsome," replied Rachael, rather puzzled to know how to answer the question.

"Ah, but he is even better than he looks," rejoined Nell. "He is so patient; he never gets tired of sitting with me, and most men hate being in a sick room. Look, he has brought me more of these yellow roses; he knows they are my favourites. Will you put them in water, and then bring them over here?"

Rachel did as Nell wished. Then, Mrs. Endsleigh coming up from dinner, Nurse Challice was free to go back to the little room next Nell's, which had been given up for her especial use.

*(To be continued.)*

